# Sequence and ionic requirements of pUG fold quadruplexes

**DOI:** 10.1080/15476286.2026.2638283

**Published:** 2026-02-27

**Authors:** Saeed Roschdi, Takuma Kume, Riley J. Petersen, Abby McCann, Cristian A. Escobar Bravo, Anika Richard, Samuel E. Butcher

**Affiliations:** Department of Biochemistry, University of Wisconsin-Madison, Madison, WI, USA

**Keywords:** RNA folding, RNAi, pUG fold, quadruplex, RNA structure

## Abstract

Poly(UG) repeats or pUG RNAs can fold into a left-handed parallel quadruplex, the pUG fold. The pUG fold directs the epigenetic amplification of RNAi in *Caenorhabditis* elegans, and pUG sequences are abundant in eukaryotic transcriptomes. Here, we report the sequence and ionic requirements for pUG folding. The pUG fold preferentially incorporates 12 guanosines but has an otherwise flexible sequence requirement. The uridines can be substituted with other nucleotides, with some sequence variants folding better than pUG RNA. The GA repeat sequence (GA)_12_ also forms a pUG-like fold, albeit with lower thermodynamic stability than (GU)_12_. The pUG fold can tolerate multiple deoxyribose substitutions but does not fold when the backbone is entirely deoxyribose. It has a high affinity and specificity for potassium ions (*K*_1/2_ = 6 mM) and does not fold in sodium or ammonium. Addition of 2 mM Mg^2+^ does not further stabilize the pUG fold, and the polyamines spermine and spermidine do not affect its stability. Finally, the pUG fold is sensitive to surrounding sequence context and complementary flanking sequences can stabilize pUG folds, while other sequences can interfere with folding. These data provide an improved ability to understand and predict pUG folds.

## Introduction

**Figure 1.**
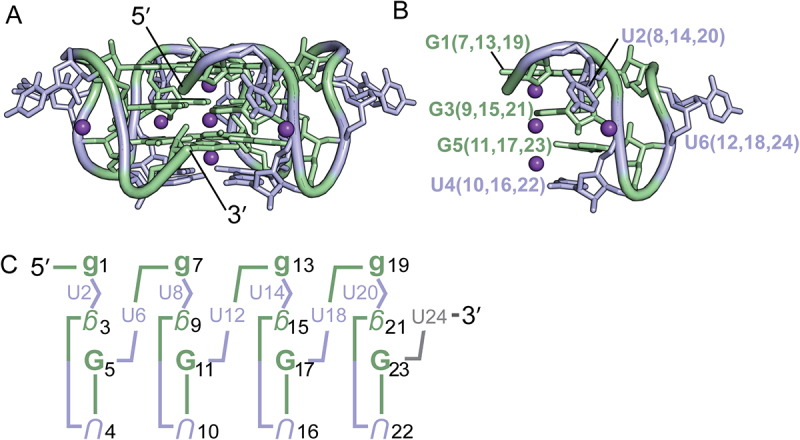
(A) Structure of the (GU)_12_ pUG Fold (PDB: 7MKT). Guanosines are shown in green, uridines in blue and potassium ions in purple (shown as one-fourth reduced size for clarity) (B) Close-up view of one-fourth of the molecule, showing the reverse S-shaped backbone of the GUGUGU hexamer, a conformation that is repeated four times. (C) Schematic of the pUG fold. Upper case is anti, lower case is syn. Backbone inversions are indicated by upside down letters. C3′ endo is italicized, C2′ endo is bold. The last 3′ uridine (grey) is disordered.

Dinucleotide repeats are common elements in eukaryotic transcriptomes, with poly(UG) or ‘pUG’ repeats being among the most abundant [1]. We previously described the pUG fold, a unique left-handed parallel intramolecular quadruplex (G4) that induces gene silencing by directing RNAi amplification in *Caenorhabditis elegans* [1,2]. The shortest sequence that can form a pUG fold is the 23-nucleotide (nt) sequence (GU)_11.5_ [1]. This sequence is extraordinarily abundant in human RNAs, occurring over 20,000 times. The polymorphic expansion of genomic GT repeats encoding pUG sequences has been associated with diseases including cancer and cystic fibrosis [1]. We determined the crystal and solution structures of the 24-nt RNA (GU)_12_, which forms a pUG fold [1,3] (Figure 1). The pUG fold has three G quartets, one U quartet and three centrally coordinated potassium ions (Figure 1). Additionally, the pUG fold has four bulged uridines, three single uridine propeller loops and, in the case of (GU)_12_, a flexible 3′ terminal uridine that is not required for the fold [1] (Figure 1). The pUG fold is distinct from other known quadruplexes as it contains no consecutive guanosines in its sequence and is the only known example of an RNA G4 with an overall left-handed topology. The left-handed topology is due to Z-form *syn-anti*-backbone inversions within the centre of the fold [1,3,4]. The 24-nt pUG fold structure has pseudo-fourfold symmetry, with four repeating units of a reverse S-shaped hexamer GUGUGU (Figure 1). Each nucleotide of the hexamer is in a different conformation with respect to glycosidic torsion angle, sugar pucker and backbone orientation (Figure 1).

RNA structures can be challenging to predict, especially for non-A-form tertiary structures [5]. For example, the pUG fold is a stable structure that forms both *in vitro* and *in vivo* [1] but is not predicted by AlphaFold 3 [6]. RNA structure prediction is complicated by the fact that even a perfectly matched sequence–structure relationship does not necessarily provide evidence for a fold. This is particularly true of flexible RNAs that are best described as ensembles of structures, the distribution of which can be influenced by the process of transcription, surrounding RNA sequence, ions, pH and the availability of small-molecule ligands and protein partners [7–9]. Regarding pUG RNA, an interesting example is found in fish, which have thousands of perfect matches to the pUG fold sequence within introns. However, these pUGs are also accompanied by complementary CA repeats, which form long-range CA/UG base-pairing interactions that bring together the 5′ and 3′ ends of introns and replace the need for the splicing factor U2AF2 [10]. On the other hand, human RNAs contain thousands of pUG repeat sequences in introns which are not accompanied by complementary CA repeats regions [1]. Humans have at least one protein that can specifically interact with pUG folds [11].

The sequence requirements and cation specificity of the pUG fold have not been described. We have previously shown that the pUG fold has some flexibility as it can tolerate AA insertions [1] and single deoxynucleotide substitutions [3]. G4 structures typically coordinate potassium ions, but some can also fold by coordinating sodium or ammonium ions [12–14]. The polyamines spermine and spermidine are also present at micromolar concentrations in human cells and can either stabilize or destabilize G4s [15–18]. It has been proposed that some RNA G4 structures may sense and regulate polyamine levels in cells [19].

Here we report the sequence and ionic requirements for pUG fold formation. We show that while 12 guanosines are incorporated into the fold, the sequence requirements are flexible and the uridines can be individually substituted by any nucleotide. Multiple U to A substitutions are allowed, and even the sequence (GA)_12_ forms a pUG-like fold. The pUG fold requires low millimolar concentrations of potassium and cannot fold in sodium or ammonium ion buffers. The presence of Mg^2+^ does not stabilize the fold, and high micromolar concentrations of polyamines do not affect its stability. The pUG fold can tolerate multiple-deoxyribose substitutions but cannot be entirely deoxyribose. Finally, we show that surrounding sequences can either stabilize or interfere with pUG folding.

## Results

### Single nucleotide variants of the pUG fold

**Figure 2.**
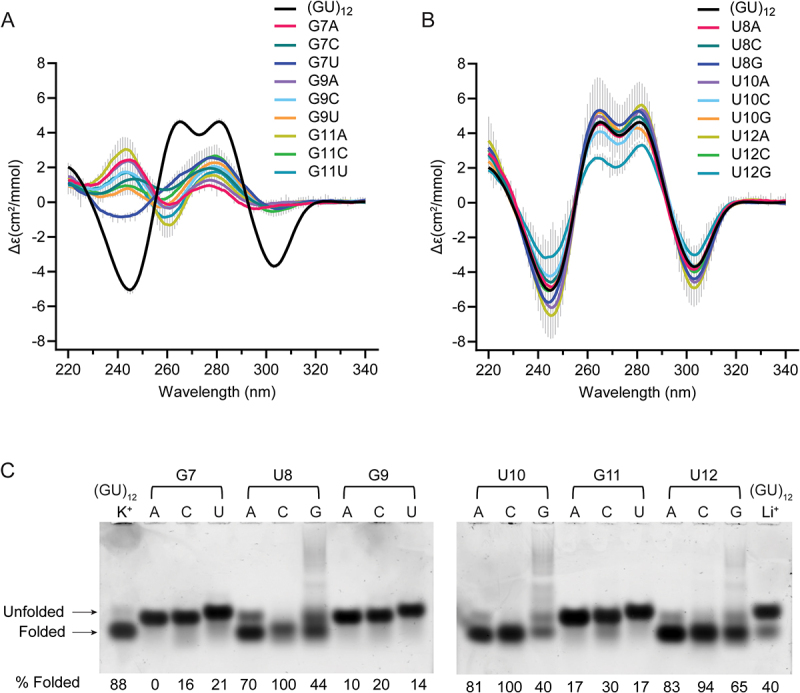
Circular dichroism spectra of single nucleotide variants that are (A) Substituted at guanosines and (B) Substituted at uridines Error bars from duplicate measurements of biological replicates are shown with vertical grey lines. (C) Native gel electrophoresis of all single nucleotide variants for positions 7–12. First and last lanes contain (GU)_12_ in K^+^ and Li^+^, respectively. Apparent percent folded is listed at the bottom of the gel.

To investigate the sequence requirements of the pUG fold, we created all possible single nucleotide variations within one of the reverse S-shaped hexameric repeats of the pUG fold (Figure 1(B)) and assessed folding via circular dichroism (CD) spectroscopy (Figure 2(A,B)). CD reports on the different base stacking geometries within the fold. The negative 244 and positive 260 nm peaks are typical of right-handed *anti–anti*-stacking in parallel quadruplexes, which is observed at (U4-G5). The positive 280 nm peak corresponds to the unusual *syn–syn* stacking observed at G1-G3, and the negative 304-nm peak corresponds to the left-handed (Z-form) *syn–anti* stacking of the G3 and G5 quartets [20]. We targeted the second hexameric repeat of the pUG fold for mutagenesis, which allowed all of the RNA variants to be efficiently transcribed *in vitro* by T7 RNA polymerase. Analysis of CD spectra for the 19 different RNAs in 150 mM KCl buffer showed that none of the G substitutions are properly folded (Figure 2(A)). In contrast to the G variants, all U variants are well folded and in fact, some fold slightly better than the reference sequence (GU)_12_ (Figure 2(B)). The intensity of the well-resolved negative CD peak at 304 nm provides an accurate and quantitative measure of the fraction of pUG folds in RNA, as it reports on the unique, left-handed conformation in the centre of the fold [1,20]. Previous measurements of pUG folding are consistent with a simple, two-state model for pUG folding [1,20], and we therefore apply that assumption here, with the caveat that different sequences may have more complex, heterogenous folding pathways and minor conformations that are not included in this model. The fraction folded and standard deviation were determined from the CD measurements of two independently prepared samples (biological replicates). The most well-folded RNA is the sequence variant U12A (Figure 2(B)). In the pUG fold, U12 is unpaired and forms a single nucleotide propeller loop that spans four quartets (Figure 1). Another well-folded sequence variant is U8G, which occurs at the bulged-out conformation within the structure (Figure 2(B)). Finally, U10 variants are folded (Figure 2(B)), and this is surprising, as this position corresponds to the U quartet. These data suggest that the U quartet can be disrupted or is flexible enough to incorporate other nucleotides. Mixed A–U–A–U quartets have been previously observed [21]. The average fraction folded for all RNAs, as determined by CD, is listed in Table 1.

**Table 1.** Folding of pUG RNA sequence variants.

Sequence	Average Fraction Folded	Standard Deviation
(GU)12	0.75	0.14
G7A	0.03	0.03
G7C	0.05	0.01
G7U	0.08	0.02
U8A	0.75	0.02
U8C	0.76	0.12
U8G	0.89	0.11
G9A	0.00	0.03
G9C	0.04	0.03
G9U	0.04	0.03
U10A	0.95	0.09
U10C	0.75	0.03
U10G	0.73	0.24
G11A	0.02	0.04
G11C	0.11	0.03
G11U	0.05	0.02
U12A	1.00	0.00
U12C	0.81	0.05
U12G	0.61	0.02
U2A	0.86	0.03
U4A	0.90	0.10
U6A	0.93	0.09
U2A, U8A, U14A, U20A	0.85	0.05
U4A, U10A, U16A, U22A	0.75	0.01
U6A, U12A, U18A, U24A	0.78	0.11
(GA)12	0.67	0.01
Random tails	0.06	0.01
AU bps	0.59	0.04
Mixed bps	0.59	0.01
Mango bps	0.50	0.03
d(GT)12	0.00	0.01
d(GU)12	0.00	0.01
dT2, dT8, dT16, dT20	0.80	0.01
dG3, dG9, dG15, dG21	0.60	0.01
d(T2, G3)x4	0.64	0.02
d(G1, T2, G3)x4	0.50	0.01
d(G1, T2, G5)x4	0.01	0.04
d(G1, T2, T6)x4	0.47	0.02
d(G1, G5, T6)x4	0.52	0.01
d(T2, G5, T6)x4	0.00	0.04
d(T4, G5, T6)x4	0.43	0.03
d(G1, T2, G5, T6)x4	0.00	0.01
d(G1, U2, G5, U6)x4	0.31	0.03
d(G1, T4, G5, T6)x4	0.12	0.01

We further analysed the sequence variants by native gel electrophoresis, in which 5 mM KCl was included in the gel and running buffer to maintain folding. In this experiment, the (GU)_12_ control RNA has a fraction folded of 0.88, which agrees with the CD data (0.75 ± 0.14) (Figure 2 and Table 1) and prior NMR-based measurements [20]. An unfolded (GU)_12_ control RNA in 150 mM LiCl partially re-folds during electrophoresis in the potassium gel and provides markers for the electrophoretic mobilities of the folded and unfolded forms of the RNA (Figure 2(C), last lane). The EMSA data confirm that the G variants are unfolded, and the U variants are folded (Figure 2(C)). An exception for the G variants is G11C, which appears to retain a small fraction of folded molecules. This agrees with the CD data showing a small negative peak for G11C corresponding to a folded fraction of 0.11 (Figure 2(A) and Table 1). These data suggest that a small fraction of molecules can incorporate a cytidine within the central G5–11–17–23 quartet of the molecule. Mixed G–C–G–C quartets have been previously observed in G4 structures [22]. The EMSA assay shows the U to A or C variants are well-folded, while the U to G variants are partially folded (Figure 2(C)), and the CD measurements show that U12G is the least well folded of the U variants (Figure 2(B)). The U to G variants display smearing in the gel, which is not observed for the other variants (Figure 2(C)). As these U to G variants create GGG stretches, we hypothesize the smearing is due to heterogenous intermolecular G4 interactions.

**Figure 3.**
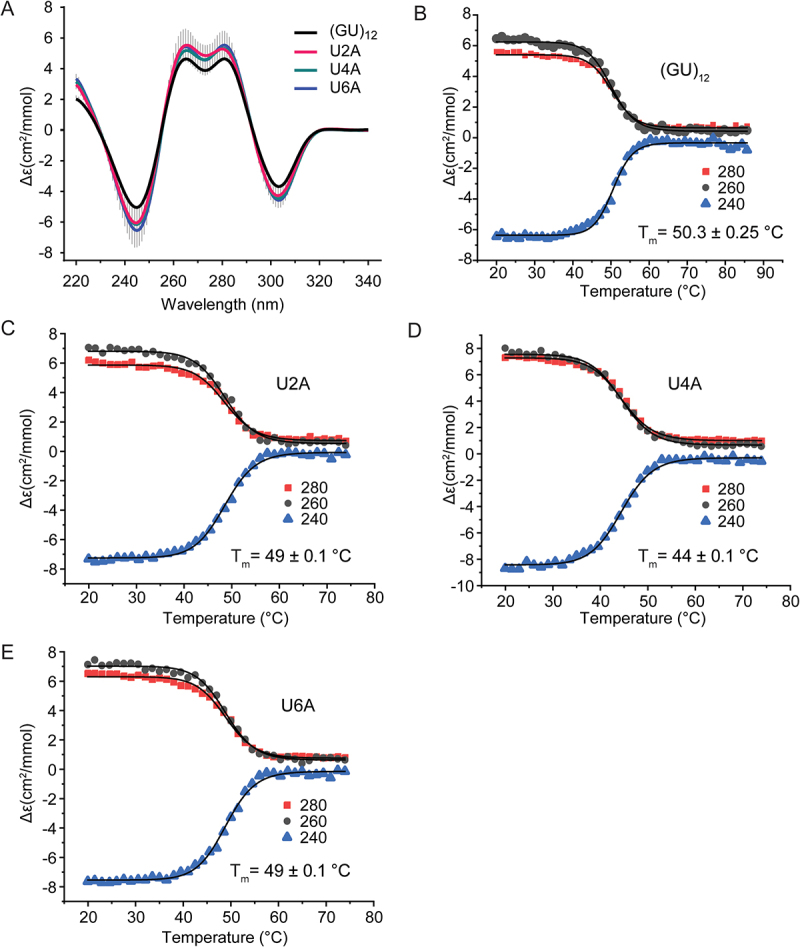
(A) Comparison of CD spectra of (GU)_12_ and U2A, U4A and U6A sequence variants. CD monitored thermal denaturation of (B) (GU)_12_, (C) U2A variant, (D) U4A variant and (E) U6A variant measured at three wavelengths. All data fitted to modified Gibbs–Helmholtz equation to determine *T_m_*.

Since the fourfold symmetry of the pUG fold is only broken by the 5′ and 3′ ends, it seems likely that sequence variations at symmetry-related positions should have similar effects on folding. To check this, we analysed U to A variants within the first hexameric repeat (U2A, U4A and U6A) (Figure 3) and compared these variants to the symmetry-related sequence variants in the second hexameric repeat (U8A, U10A and U12A) (Figure 2). Indeed, the resulting data are similar and within the standard deviation of the measurements for all but U2A and U8A. The U2A, U4A and U6A have folded fractions of 0.86, 0.90 and 0.93, respectively, while the symmetry-related variants U8A, U10A and U12A have folded fractions of 0.75, 0.95 and 1.0, respectively (Table 1).

We further analysed the thermodynamic stabilities of the U to A variants (U2A, U4A, and U6A) (Figure 3). Temperature melting data were fit to the modified Gibbs–Helmholtz equation to determine the *T_m_* [23]. The reference sequence (GU)_12_ has a *T_m_* = 50.3°C, close to our previously measured value of 51.5°C for (GU)_11.5_ [1]. The U2A and U6A variants have similar thermodynamic stabilities, with *T_m_* = 49°C (Figure 3(C,E)). In contrast, a decrease in stability is observed for the U-quartet variant U4A, with *T_m_* = 44°C (Figure 3(D)). Therefore, although substitutions in the U quartet are tolerated, the U quartet adds stability to the overall fold.

### Extensive sequence variants of the pUG fold

**Figure 4.**
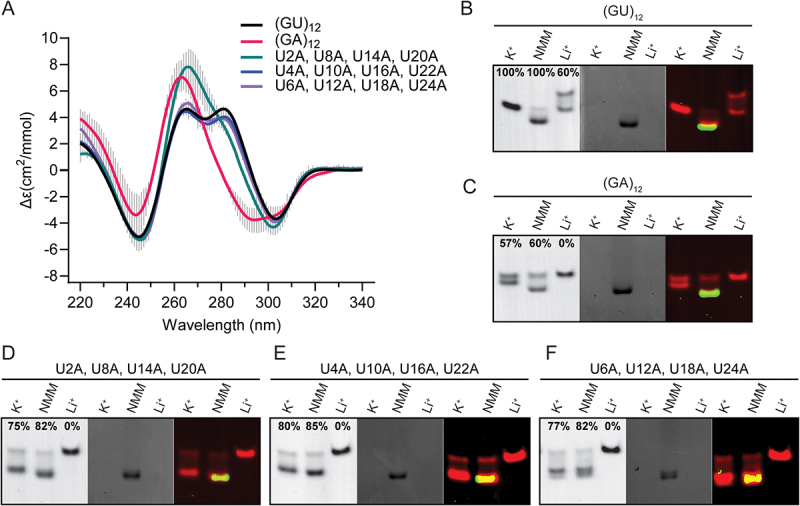
(A) CD spectra of pUG folds with multiple a substitutions including quadruple U to a variants and (GA)_12_. (B–F) Native gel electrophoresis of RNAs folded in K^+^ in the presence and absence of NMM, compared to unfolded RNA after Li^+^ treatment. RNAs were visualized using toluidine blue staining (left), NMM fluorescence (middle), and with both images overlayed in false colors (right). Data are shown for (B) (GU)_12_, (C) (GA)_12_, (D) U2A, U8A, U14A, U20A, (E) U4A, U10A, U16A, U22A and (F) U6A, U12A, U18A and U24A.

To further investigate the sequence flexibility of the pUG fold, we measured the folding of sequence variants containing multiple U to A substitutions (Figure 4(A)). These variants included quadruple U to A substitutions at a given position in each of the four hexameric repeat units, and the sequence (GA)_12_. All RNAs were folded and display the negative 304 nm peak characteristic of the Z-form *syn–anti* stacking of the central G3 and G5 quartets (Figure 4(A)). The quadruple bulged nucleotide variant (U2A, U8A, U14A and U20A) has a much smaller 280-nm peak but is otherwise folded (Figure 4(A)). The 280-nm peak corresponds to the *syn–syn* stacking conformation of the G1–7–13–19 and G3–9–15–21 quartets, and the (U2A, U8A, U14A, U20A) bulge variants occur between these quartets. Therefore, adenosines at this bulge position may alter the stacking geometry of the G1 and G3 quartets while leaving the rest of the fold intact. The quadruple U to A variant at the U quartet (U4A, U10A, U16A, U22A) is folded and displays all four peaks associated with the pUG fold (Figure 4(A)). These data further demonstrate that the U quartet is not necessary for pUG folding. This is likely because the U4 quartet is located at a solvent exposed edge of the structure and only forms four hydrogen bonds, vs. the G quartets which have eight hydrogen bonds [1,3]. The quadruple U to A propeller loop variant (U6A, U12A, U18A and U24A) is also well folded (Figure 4(A) and Table 1). These data demonstrate that the pUG fold can tolerate multiple U to A substitutions at any position in the fold. We therefore asked if all uridines can be replaced by adenosine and tested the folding of (GA)_12_. Similar to the bulged variant (U2A, U8A, U14A, U20A), the (GA)_12_ variant displays a pUG-like fold that is missing the 280 nm peak but is otherwise folded (Figure 4(A) and Table 1).

To further visualize the folding of these sequence variants, we analysed them by EMSA and tested them for the ability to bind to *N*-methyl-mesoporphyrin (NMM), a G4 ligand that binds to the pUG fold with a *K*_D_ of 1 μM by stacking on the G1 quartet and forming hydrogen bonds with 2′ hydroxyl groups [1]. In these experiments, NMM was added after the RNA folding protocol, just prior to electrophoresis. When bound to NMM, the pUG fold migrates more quickly through the gel, likely due to the two negatively charged carboxylate groups on NMM (Figure 4(B–F)). All variants display potassium-dependent electrophoretic mobilities identical to the pUG fold and are unfolded when treated with lithium (Figure 4(B–F)). Additionally, all variants bind to NMM, consistent with G4 formation (Figure 4(B–F)). NMM fluorescence increases 60-fold when stacked upon G4s [1,24], and all variants showed this enhanced NMM fluorescence upon binding (Figure 4(B-F)). The (GA)_12_ RNA is folded as measured by CD (Figure 4(A) and Table 1) and is mostly folded in the EMSA assay (Figure 4(C)). The EMSA data show that the folded conformation of (GA)_12_ binds NMM, as expected for a G4 (Figure 4(C)). The quadruple U to A variants are well-folded in both the CD and EMSA assay and all bind NMM (Figure 4(D–F) and Table 1). For (GA)_12_ and all U to A variants, NMM binding slightly increases the folded fraction of RNA, by 3–7% (Figure 4(C–F)). This is consistent with a stabilizing effect of NMM. NMM binding modestly stabilizes the pUG fold, increasing its melting temperature from 51.5 to 59.7°C [1]. In general, there is a good correlation between the CD and EMSA measurements of the fraction folded for all RNAs (Supplemental Figure 1).

We measured the thermodynamic stabilities of the quadruple U to A substitutions and the (GA)_12_ variant, which have low melting temperatures of 32–36°C (Supplemental Figure 2 and Supplemental Table 1). Therefore, multiple U to A substitutions significantly destabilize the pUG fold. Nevertheless, all variants can adopt pUG-like folds which include the characteristic left-handed *syn–anti* stacking interactions within the centre of the fold. We therefore define ‘pUG-like’ folds as G4 structures with left-handed topologies that result from Z-form like *syn–anti* stacking within the fold, which can be readily observed as a distinctive negative peak at 304 nm in CD spectra. The 304-nm peak is not found in a CD library of 23 other quadruplex topologies [25], and to our knowledge this signal has thus far only been observed for the pUG fold and Z-form RNA stabilized by high ionic strength [26].

**Figure 5.**
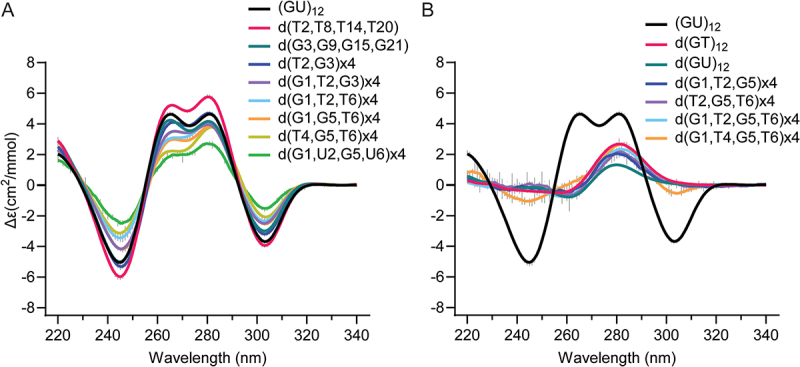
CD spectra of (A) deoxy substitutions that are folded and (B) deoxy substituted variants that are unfolded. For reference, the (GU)_12_ pUG Fold is shown in black. Variants designated x4 include deoxy substitutions at all symmetry-related positions.

We next tested whether the pUG fold can tolerate multiple deoxyribose substitutions. Our previous NMR data showed that single deoxyribose substitutions are tolerated by the pUG fold at each of the six different positions in the first hexameric repeat [3]. Four out of the six nucleotide conformations in the hexamer (positions 1, 2, 5 and 6) have 2′ endo sugar puckers, which is favoured by deoxyribose [1,3]. However, the 2′ hydroxyl groups of the second and third nucleotides form water-mediated hydrogen bonds to a partially hydrated K^+^ ion [1] (Figure 1(A,B)). We therefore hypothesized that the pUG fold may tolerate deoxyribose substitution at positions which are 2′ endo but do not form known ionic interactions, which correspond to positions 1, 5 and 6 of each hexamer. Indeed, the d (G1, G5, T6)x4 variant is folded (Figure 5(A)), although not as well as (GU)_12_ (0.52 vs. 0.75, respectively) (Table 1). Deoxythymidine substitution is well tolerated at the second position, and the d(T2, T8, T14, T20) variant folds better than (GU)_12_ (Figure 5(A) and Table 1). The pUG fold cannot be entirely DNA, as neither d(GU)_12_ or d(GT)_12_ fold (Figure 5(B)). Some variants with 50–66% deoxyribose are partially folded (Figure 5(A)), while others are unfolded (Figure 5(B)). These data indicate that the pUG fold can tolerate partial but not complete deoxyribose substitution.

### Ionic requirements for pUG Fold formation

**Figure 6.**
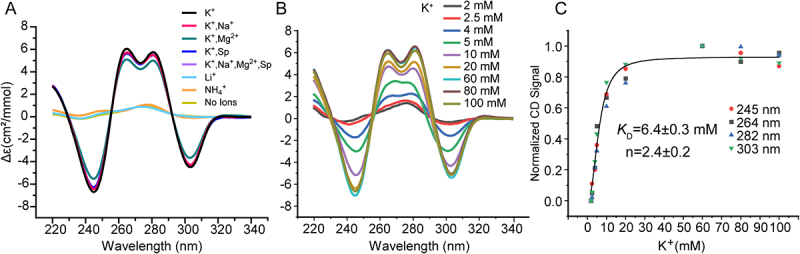
(A) (GU)_12_ RNA folding in buffers with different cations including: 150 mM K^+^, 150 mM K^+^ and 2 mM Mg^2+^, 140 mM K^+^, 10 mM Na^+^, 2 mM Mg^2+^, 0.3 mM spermine and 0.4 mM spermidine (K^+^ + Mg^2+^+Na^+^ + SP), 150 mM Na^+^, 150 mM Li^+^, 150 mM NH_4_^+^, and no ions (20 mM tris buffer pH 7.0). (B) K^+^ concentration dependence of pUG folding monitored by CD at specified K^+^ concentrations. All samples were 20 mM RNA. (C) Normalized CD data from (B) fit to the Hill equation.

We investigated the cation dependence of pUG folding in potassium, ammonium and sodium containing buffers. We find the pUG fold specifically requires potassium ions and cannot fold in 150 mM sodium or ammonium buffers (Figure 6(A)). We also investigated pUG folding in 150 mM KCl and 2 mM MgCl_2_, and in a pseudo-physiological buffer condition with mixed cations (140 mM KCl, 10 mM NaCl, 2 mM MgCl_2_, 0.3 mM spermine and 0.4 mM spermidine) [15] (Figure 6(A)). We titrated potassium and fit the resulting CD data to the Hill equation to determine the apparent equilibrium folding constant (*K*_0.5_ = 6.4 mM) and the Hill coefficient (*n* = 2.4) for potassium (Figure 6(B,C)). The Hill coefficient is consistent with cooperative uptake of multiple potassium ions, as observed in the structure. We performed CD temperature melting experiments to compare thermodynamic stabilities and observed no difference between the potassium only (*T_m_* = 50.3°C) and potassium with magnesium (*T_m_* = 50.6°C) (Supplemental Figure 3). These data indicate that magnesium does not stabilize the pUG fold. We also do not observe any difference in stability in 140 mM K^+^ and addition of 10 mM Na^+^, 2 mM Mg^2+^, 0.3 mM spermine and 0.4 mM spermidine, or a combination of all these cations in the pseudo-physiological buffer (*T_m_* = 50.3°C) (Supplemental Figure 3). Polyamines have been previously observed to destabilize quadruplexes with short loops [15] but do not affect the stability of the pUG fold.

### Flanking sequences affect pUG folding

**Figure 7.**
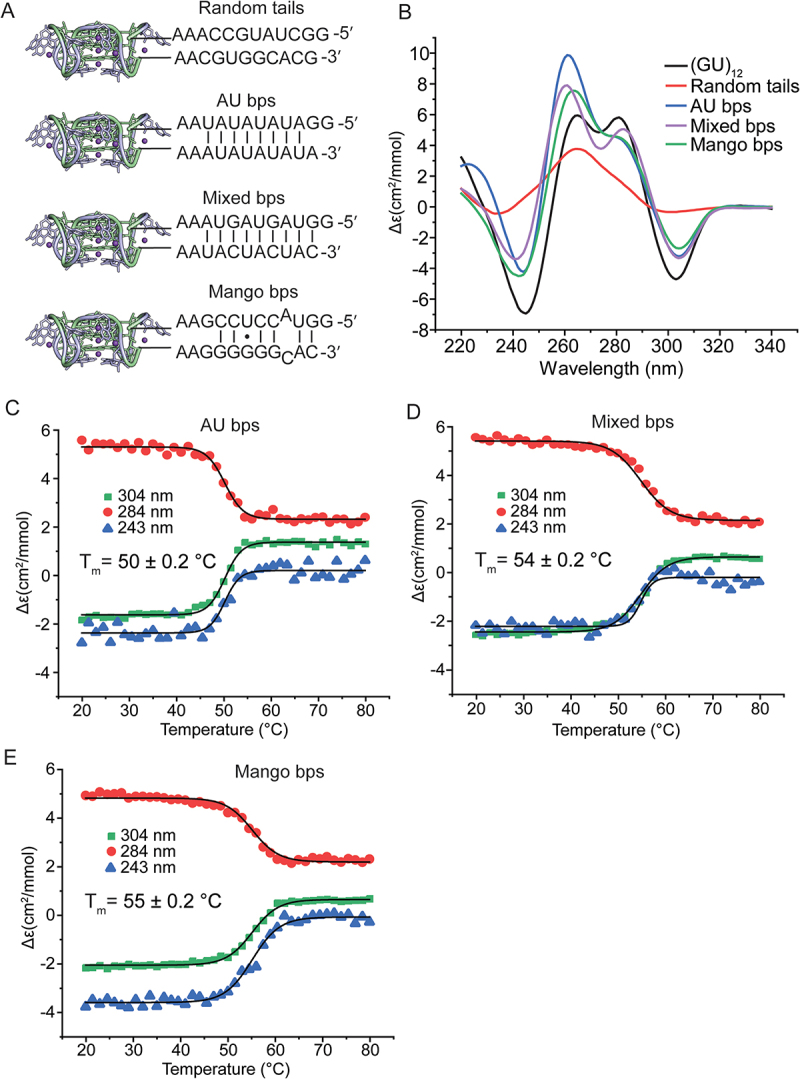
(A) 47 nucleotide RNAs with indicated 5′ and 3′ flanking sequences attached to (GU)_12_. (B) CD spectra of RNAs. (C–E) thermal melts of (C) AU bps RNA, (D) mixed bps and (E) mango bps constructs.

We previously showed that the pUG fold can form in the middle of an RNA when flanked by polyadenosines [1] and at RNA 3′ ends [3]. To investigate the effect of structured flanking sequences on pUG folding, we created four different RNA constructs, each 47 nt in length. We reasoned that complementary flanking sequences might stabilize the pUG fold, similar to how G4-containing fluorogenic aptamers are stabilized by adjacent A-form helices [27–31]. All sequences contained AA linkers between the pUG fold and the flanking sequences (Figure 7), similar to the fluorogenic aptamers [27–31]. As a control, we created a construct with randomly generated 5′ and 3′ flanking sequences with little to no predicted base pairing between these flanking sequences (Figure 7(A), random tails). Three additional constructs were generated with base pairing between the flanking sequences: one with AU base pairs (bps), one with mixed bps and one with bps based on the original mango aptamer sequence [27] (Figure 7(A)). All constructs folded except for the random tail sequence (Figure 7(B)). CD spectra of A-form helices are characterized by a positive peak at 260 nm [32]. An increase in the intensity of the 260-nm peak is observed for the AU bps, mixed bps and mango bps sequences, consistent with the formation of pUG folds with additional A-form secondary structures. We have previously shown that the fraction of folded pUG folds can be calculated from the intensity of the negative 304-nm peak, with the maximum intensity observed when all nucleotides are in a pUG fold [20]. In the case of the AU bps, mixed bps and mango bps constructs, the pUG fold engages 24 out of 47 nucleotides, and complete pUG fold formation as measured by the change in molar ellipticity at 304 nm should produce a normalized fraction of pUG folded nucleotides of ~24/47, or 0.51. Analysis of the CD data for the AU bps, mixed bps and mango bps sequences shows that indeed, these RNAs have pUG-folded fractions very close to 0.51 (Table 1). This in turn suggests that the AU bps, mixed bps and mango bps RNA constructs are nearly completely folded, as the molar ellipticity is consistent with simultaneous formation of A-form helices and complete pUG fold formation for 24/47 nucleotides.

To further understand the folding of these RNAs, we performed RNA secondary structure predictions using energy minimization based on nearest-neighbour free energies using the RNAStructure version 6.5 online webserver with default settings [33]. These predictions show the mixed bp and mango tail constructs have ‘unpaired’ pUG regions that are available for pUG folding (Supplemental Figure 4). The secondary structure prediction for the AU bp construct pairs the pUG sequence in a way that precludes pUG folding, which is clearly incorrect and highlights inherent challenges with predicting quadruplexes (Supplemental Figure 4). However, the prediction for the random tail construct aligns with the CD data, as this RNA does not adopt a pUG fold. The random tail construct included the di- and trinucleotide sequences CA and CAC, which are perfectly complementary to pUGs, and allows formation of a stem-loop structure that likely prevents pUG folding (Supplemental Figure 4).

We measured the thermodynamic stabilities of the pUG folds in the mango, AU bp and mixed bp tail constructs and compared them to the isolated (GU)_12_ pUG fold. The pUG folds in the mango tails and mixed bp tail RNAs are more stable than the isolated pUG fold (T_m_ = 54–55 vs. 52°C) while the AU-rich tail pUG fold was slightly destabilized (*T_m_* = 50°C) (Figure 7). The observation that the mango tails and mixed bp tails constructs are more thermodynamically stable than the isolated pUG fold is consistent with the flanking helices having a stabilizing effect on the pUG fold. In contrast, other flanking sequences can destabilize or interfere with folding, especially if they contain CA-rich regions that are complementary to pUGs. Thus, pUG fold formation and stability are highly dependent on sequence context.

### Discussion

Here, we show that the pUG fold is not limited to poly(UG) RNA. We systematically tested a total of 40 sequence variants for pUG folding (Table 1). From these data, we ascertain that the pUG fold consensus sequence is flexible aside from a requirement for 12 guanosines. The spacing of the 12 guanosines is also flexible, as we have previously shown that the pUG fold can tolerate AA insertions [1]. Our data indicate that any single uridine in the fold can be substituted by any other nucleotide. Single uridine substitutions and multiple U to A substitutions are well tolerated. U to G variants adopt pUG folds but show some degree of misfolding that can be attributed to the GGG stretches produced by these variants, which likely induce heterogenous G4 interactions. Additionally, multiple uridines cannot be substituted with cytidine, as this would create self-complementary GC-rich sequences that form stable hairpin or duplex structures. We speculate that as few as two or three U to C substitutions, depending upon their position in the sequence, may result in alternative secondary structures with CG base pairs that prevent pUG folding. Indeed, the formation of three C–G base pairs can effectively prevent pUG folding (Figure 7 and Supplemental Figure 3).

To our knowledge, this is the first report of GA repeat RNA forming left-handed G4 structures. GA repeat RNAs have been found to be associated with replicated DNA [34]. The idea that RNAs with GU and GA repeats may form similar G4 structures is consistent with previous work showing that both GU and GA repeats inhibit ribosome scanning and induce frameshifting but only in the presence of G4-stabilizing ligands [35]. While the (GA)_12_ structure forms a pUG-like fold at room temperature, its thermodynamic stability is significantly lower than the pUG fold (32 vs. 52°C). We find that four U to A substitutions is enough to significantly decrease the stability of the pUG fold (*T_m_* = 34–36°C). Since the primary source of nucleic acid thermodynamic stability is base stacking, destabilization via adenosine substitution is likely due to adenosine stacking interactions that disrupt the fold. This is consistent with the observation that adenosine substitution at U2 and symmetry-related positions disrupts G1-G3 stacking, as evidenced by loss of the corresponding 280 nm CD peak (Figure 4(A)). In contrast, adenosine substitution at the U quartet, which is on a solvent exposed edge of the structure, preserves the complete CD spectrum but still destabilizes the fold, likely via disruption of K^+^ ion coordination that is maintained in part by the U quartet carbonyl oxygens (Figure 1(A,B)). Thus, GA-rich sequences may require stabilization by ligands or additional folded elements to adopt pUG folds at physiological temperatures. Ligands that can stabilize pUG folds include small molecules such as porphyrins [1] and proteins. The human protein DNMT1 is one such pUG fold binding protein, and RiP-Seq experiments show that it binds to diverse sequences that are enriched with UG repeats [11].

While the pUG fold does not form in DNA, DNA GA repeats have been previously observed to form four stranded structures [36–38]. DNA GT repeats have also been hypothesized to form G4 structures under conditions of extreme molecular crowding [39]. However, we find no evidence for G4 formation by DNA GT repeats in the absence of molecular crowding. We reason that DNA does not form pUG folds due to a combination of effects that include incorrect sugar puckers and loss of interactions between 2′ hydroxyl groups and partially hydrated K^+^ ions (Figure 1(A)and (B)).

The pUG fold has a high affinity and specificity for potassium ions, unlike other quadruplexes that have more relaxed ionic requirements and can form in potassium as well as sodium or ammonium [38,40–44]. The pUG fold’s selectivity for potassium ions can be attributed to the optimal fit of the ion, as observed in previous high-resolution structures of pUG G4s in mixed K^+^ and Na^+^ salts [45], and may also relate to the greater energetic cost of sodium ion dehydration [46], or a combination of these effects. The *K*_0.5_ of 6 mM K^+^ and *n* = 2.4 values measured here are within the range of observed values for other G4 structures [47]. Highlighting the pUG fold’s selectivity for K^+^ is the fact that its stability is unaffected by the addition of physiological concentrations of Mg^2+^, which is unusual for an RNA structure. This may be due to the fact that the pUG fold binds six potassium ions, an average of one cation for every four nucleotides (Figure 1) [1]. Although Mg^2+^ does not affect the melting temperature of the pUG fold, it does accelerate its folding kinetics [20].

The data presented here expand our understanding of nucleic acid folding and demonstrate that diverse, commonly occurring RNA sequences can form pUG folds. Currently, it is not possible to predict pUG fold formation, as further improvements in RNA secondary and 3D structure prediction methods are needed and this remains a challenging area of computational biology. While the human transcriptome contains >20,000 perfect sequence matches to the (GU)_12_ pUG fold sequence [1], we find that there are additional ~4100 pUG sequence variants with single U to N substitutions that can potentially adopt pUG folds (Supplemental Figure 5). The human transcriptome also contains ~2400 GA repeats with at least 12 Gs (Supplemental Figure 5). Taken together as an upper limit, we estimate there are ~27,000 sequences that could potentially adopt pUG folds within the human transcriptome, which is more than the number of human genes. However, we also show that pUG folds are highly sensitive to flanking sequences that can either stabilize or destabilize the fold. It therefore seems likely that a prerequisite for pUG fold formation is the absence of competing high-probability secondary structures. On the other hand, pUG fold formation is favoured when the surrounding sequences are complementary. Base pairing of complementary sequences prevents competing interactions, while simultaneously bringing the 5′ and 3′ ends into proximity, which is a requirement for the pUG fold (Figure 1(A)). In *C. elegans*, pUG tails are added to RNA 3′ ends and can be over 100 nt long [2]. The formation of pUG folds in *C. elegans* is a requirement for the recruitment of RNA-dependent RNA polymerase for the synthesis of secondary siRNAs [1]. Long pUGs at 3′ ends likely provide an optimal context for pUG folding *in vivo*, as there are no neighbouring sequences to interfere with folding. Given the large number of potential pUG fold sequences within genomes, it will be interesting in the future to determine when and where they form in other organisms. More broadly, a quantitative framework is needed for improved RNA secondary prediction tools that incorporate the energetics of quadruplexes and other folds.

## Methods

### RNA synthesis

RNAs were transcribed using T7 RNA polymerase and purified on 15% denaturing polyacrylamide gel electrophoresis (7 M urea, 100 mM tris, 90 mM boric acid, 10 mM EDTA), as previously described [48,49]. The RNAs were identified using UV-shadowing and removed from the gel using a razor blade followed by crushing and soaking at room temperature overnight in 300 mM sodium acetate pH 5.6 and 1 mM EDTA. RNA solutions were then passed through a 0.2-μm filter and further purified using a 1-ml Hi-trap *Q* column (GE Healthcare) that was pre-equilibrated in 100 mM NaCl, 10 mM KH_2_PO_4_, 10 mM K_2_HPO_4_ and 1 mM EDTA, pH 7.2. RNA was washed with 20 ml of buffer, then eluted with 2 M NaCl, 10 mM KH_2_PO_4_, 10 mM K_2_HPO_4_ and 1 mM EDTA, pH 7.2. RNAs were concentrated in an Amicon Ultra 3-kDa centrifugal filter and extensively buffer-exchanged into nuclease-free distiled water (Invitrogen). During buffer exchange, the samples were concentrated 30-fold from 3 ml to 0.1 ml, to which 2.9 ml of nuclease-free water was added, and this procedure was repeated 3 times. The concentrated purified RNAs in water were then further diluted ~25-fold into the desired buffers. From the dilution factors, we calculate that the RNAs in potassium and ammonium containing buffers also contained micromolar amounts of residual sodium ions carried over during purification. All RNA samples were pre-folded by in 20 mM bis–tris pH.7 buffer and the appropriate salts by heating in 1 l of 90°C water and allowing the water bath to slowly cool to room temperature for 5–6 h.

### RNA oligonucleotides used in this study

(GU)_12_: 5′-GUGUGUGUGUGUGUGUGUGUGUGU-3′

G7A: 5′-GUGUGUAUGUGUGUGUGUGUGUGU-3′

G7C: 5′-GUGUGUCUGUGUGUGUGUGUGUGU-3′

G7U: 5′-GUGUGUUUGUGUGUGUGUGUGUGU-3′

U8A: 5′-GUGUGUGAGUGUGUGUGUGUGUGU-3′

U8C: 5′-GUGUGUGCGUGUGUGUGUGUGUGU-3′

U8G: 5′-GUGUGUGGGUGUGUGUGUGUGUGU-3′

G9A: 5′-GUGUGUGUAUGUGUGUGUGUGUGU-3′

G9C: 5′-GUGUGUGUCUGUGUGUGUGUGUGU-3′

G9U: 5′-GUGUGUGUUUGUGUGUGUGUGUGU-3′

U10A: 5′-GUGUGUGUGAGUGUGUGUGUGUGU-3′

U10C: 5′-GUGUGUGUGCGUGUGUGUGUGUGU-3′

U10G: 5′-GUGUGUGUGGGUGUGUGUGUGUGU-3′

G11A: 5′-GUGUGUGUGUAUGUGUGUGUGUGU-3′

G11C: 5′-GUGUGUGUGUCUGUGUGUGUGUGU-3′

G11U: 5′-GUGUGUGUGUUUGUGUGUGUGUGU-3′

U12A: 5′-GUGUGUGUGUGAGUGUGUGUGUGU-3′

U12C: 5′-GUGUGUGUGUGCGUGUGUGUGUGU-3′

U12G: 5′-GUGUGUGUGUGGGUGUGUGUGUGU-3′

U2A: 5′-GAGUGUGUGUGUGUGUGUGUGUGU-3′

U4A: 5′-GUGAGUGUGUGUGUGUGUGUGUGU-3′

U6A: 5′-GUGUGAGUGUGUGUGUGUGUGUGU-3′

U2A, U8A, U14A, U20A: 5′-GAGUGUGAGUGUGAGUGUGAGUGU-3′

U4A, U10A, U16A, U22A: 5′-GUGAGUGUGAGUGUGAGUGUGAGU-3′

U6A, U12A, U18A, U24A: 5′-GUGUGAGUGUGAGUGUGAGUGUGA-3′

(GA)_12_: 5′-GAGAGAGAGAGAGAGAGAGAGAGA-3′

Random tails: 5′-GGCUAUGCCAAAGUGUGUGUGUGUGUGUGUGUGUGUAACGUGGCACG-3′

AU tails: 5′-GGAUAUAUAUAAGUGUGUGUGUGUGUGUGUGUGUGUAAAUAUAUAUA-3′

Mixed bp tails: 5′-GGUAGUAGUAAAGUGUGUGUGUGUGUGUGUGUGUGUAAUACUACUAC-3′

Mango tails: 5′-GGUACCUCCGAAGUGUGUGUGUGUGUGUGUGUGUGUAAGGGGGGCAC-3′

d(GT)_12_: 5′-dGdTdGdTdGdTdGdTdGdTdGdTdGdTdGdTdGdTdGdTdGdTdGdT-3′

d(GU)_12_: dGdUdGdUdGdUdGdUdGdUdGdUdGdUdGdUdGdUdGdUdGdUdGdU-3′

d(T2,T8,T14,T20): 5′-GdTGUGUdTGUGUGdTGUGUGdTGUGU-3′

d(G3,G9,G15,G21): 5′-GUdGUGUGUdGUGUGUdGUGUGUdGUGU-3′

d(T2,G3)x4: 5′-GdTdGUGUGdTdGUGUGdTdGUGUGdTdGUGU-3′

d(G1,T2,G3)x4: 5′-dGdTdGUGUdGdTdGUGUdGdTdGUGUdGdTdGUGU-3′

d(G1,T2,G5)x4: 5′- dGdTGUdGUdGdTGUdGUdGdTGUdGUdGdTGUdGU-3′

d(G1,T2,T6)x4: 5′-dGdTGUGdTdGdTGUGdTdGdTGUGdTdGdTGUGdT-3′

d(G1,G5,T6)x4: 5′-dGUGUdGdTdGUGUdGdTdGUGUdGdTdGUGUdGdT-3′

d(T2,G5,T6)x4: 5′-GdTGUdGdTGdTGUdGdTGdTGUdGdTGdTGUdGdT-3′

d(T4,G5,T6)x4: 5′-GUGdTdGdTGUGdTdGdTGUGdTdGdTGUGdTdGdT-3′

d(G1,T2,G5,T6)x4: 5′-dGdTGUdGdTdGdTGUdGdTdGdTGUdGdTdGdTGUdGdT-3′

d(G1,U2,G5,U6)x4: 5′-dGdUGUdGdUdGdUGUdGdUdGdUGUdGdUdGdUGUdGdU-3′

d(G1,T4,G5,T6)x4: 5′-dGUGdTdGdTdGUGdTdGdTdGUGdTdGdTdGUGdTdGdT-3′

### RNA folding analyses by native gel electrophoresis

Samples for native gel folding analyses were 10 μM RNA. To the indicated samples, 10 μM *N*-methyl mesoporphyrin IX (NMM) was added. RNA samples were mixed with an equal volume of 40% sucrose and loaded onto a 1 mm-thick native 10% native polyacrylamide gel (29:1 acrylamide:bis acrylamide) containing KCl and Tris–borate–EDTA buffer (100 mM Tris, 90 mM Boric acid, 10 mM EDTA, 5 mM KCl) and were run at 4°C for 2 h at 5 W. Gels were stained with 0.1% toluidine blue dye or SYBR Gold (Thermo Fisher Scientific). Gels were imaged using a CanoScan LIDE300 image scanner (Canon) or Amersham Typhoon 5 gel scanner (Cytiva). Fluorescent NMM samples were imaged with 488 and 670 nm wavelength filters for excitation and emission, respectively.

### Circular dichroism (CD) spectroscopy

CD samples contained 20 μM RNA in 20 mM Tris buffer pH 7.0 and either 150 mM KCl or 150 mM LiCl. RNA samples for melting temperature experiments contained 20 μM RNA in 20 mM potassium phosphate buffer pH 7.0 and 130 mM KCl. CD spectra were recorded in an AVIV model 420 CD spectrometer using a quartz cell with an optical path length of 1 mm. The scans were carried out with a step size of 1 nm and all data points were signal averaged for 5 s. CD measurements were taken from 210 to 340 nm at 25°C. Buffer subtraction was performed and data were converted to molecular CD values, Δ*ε*:

$$\Delta \varepsilon =\frac{\theta }{32,980\times C\times L\times N}$$

where *θ* is the raw CD signal (in millidegrees), 32,980 is the standard conversion factor for expressing CD signal in millidegrees, *C* is the RNA concentration (in M), *L* is the cuvette path length (in cm), and *N* is the number of nucleotides. Thermal denaturation studies were also carried out by heating each sample from 20 to 85°C in 1.5°C intervals and with a 5-min equilibration time at each temperature. The ellipticity was measured at four different wavelengths (244, 264, 284 and 340 nm) with an averaging time of 10 s at each temperature. The thermal unfolding profile was characterized by the millidegree signal at 244, 264 and 284 nm to determine *T*_*m*_ and Δ*G* (37°C) values by global fitting of the data to the Modified Gibbs–Helmholtz equations using Origin 2020 (OriginLab Corporation) [23]. The potassium dependence of RNA folding was determined by collecting full wavelength scans as above. The 244, 264, 282, and 304 nm peaks were normalized with the maximum CD signal set to 1 to determine the equilibrium folding constant (*K*_0.5_) and hill coefficient (*n*) by globally fitting the data to the Hill equation using Origin (Origin 2020 OriginLab Corporation).

### Genome analysis

Human genome analysis was performed using the source sequence: NCBI RefSeq assembly GCF_000001405.40 (GRCh38.p14), annotation GCF_000001405.40–RS_2025_08. The search pattern code used is freely available https://gitlab.com/TakumaKawajiKume/rust-anyU_pUG. Briefly, this code searches for ‘GN’ repeats that are >23 nucleotides, with overlapping sequences counted as one sequence. The code provides the information for which nucleotides deviate from the canonical pUG repeat sequence, total number of deviations, and overlapping annotations that are in the same strand direction.

## Supplementary Material

Roschdi_Kume_Supplemental.pdf

## Data Availability

All data will be made available on reasonable request. Circular dichroism source data are available at https://doi.org/10.6084/m9.figshare.30460727
