# Supplementary material for: Sequence and ionic requirements of pUG fold quadruplexes
Source: RNA Biol. 2026 Feb 27;23(1):1–16. doi: 10.1080/15476286.2026.2638283 (PMC12962679; doi:10.1080/15476286.2026.2638283)
Supplement: Roschdi_Kume_Supplemental.pdf [file KRNB_A_2638283_SM5530.pdf]

Supplemental Data

Sequence and ionic requirements of pUG fold quadruplexes

Saeed Roschdi[1], Takuma Kume[1], Riley J. Petersen, Abby McCann, Cristian A. Escobar, Anika Richard and Samuel E. Butcher*

[1]Equal contribution
Department of Biochemistry, University of Wisconsin-Madison, Madison, WI, USA.
*Correspondence: sebutcher@wisc.edu

Supplemental Table 1

Sequence	T_m °C
$(GU)_{12}$ (150 mM K^+)	52
$(GU)_{12}$ (150 mM K^+, 2 mM Mg^{2+})	51
$(GU)_{12}$ (K^+, Na^+, Mg^{2+}, Sp)	45
U2A	49
U4A	44
U6A	49
U2A, U8A, U14A, U20A	36
U4A, U10A, U16A, U22A	35
U6A, U12A, U18A, U24A	34
$(GA)_{12}$	32
AU bps	50
Mixed bps	54
Mango bps	55

Supplemental Figure 1

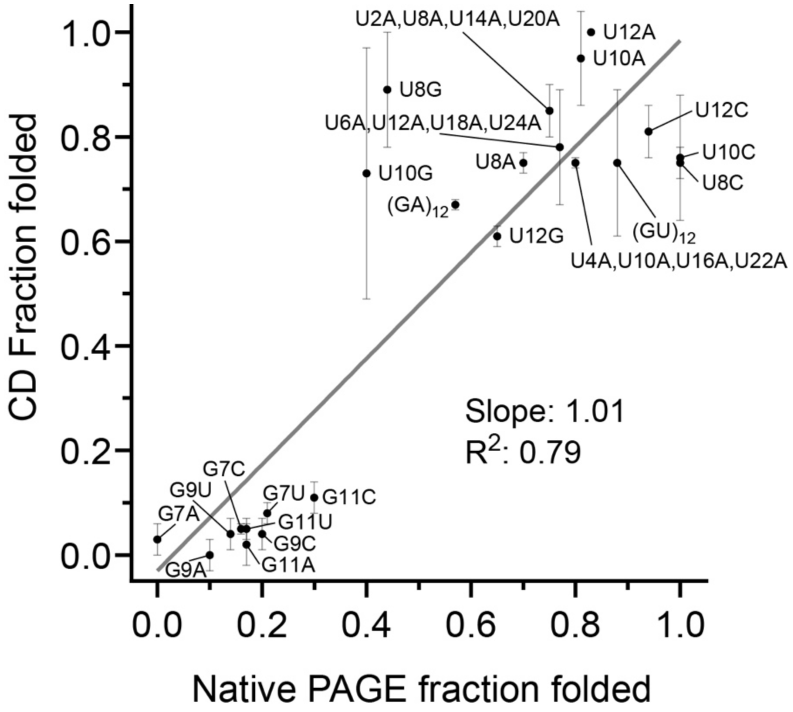

Supplemental Figure 1. Comparison of fraction folded determined by CD vs. native page. Note CD measurements were performed in 150 mM KCl, while native page experiments were performed in 5 mM KCl.

Supplemental Figure 2

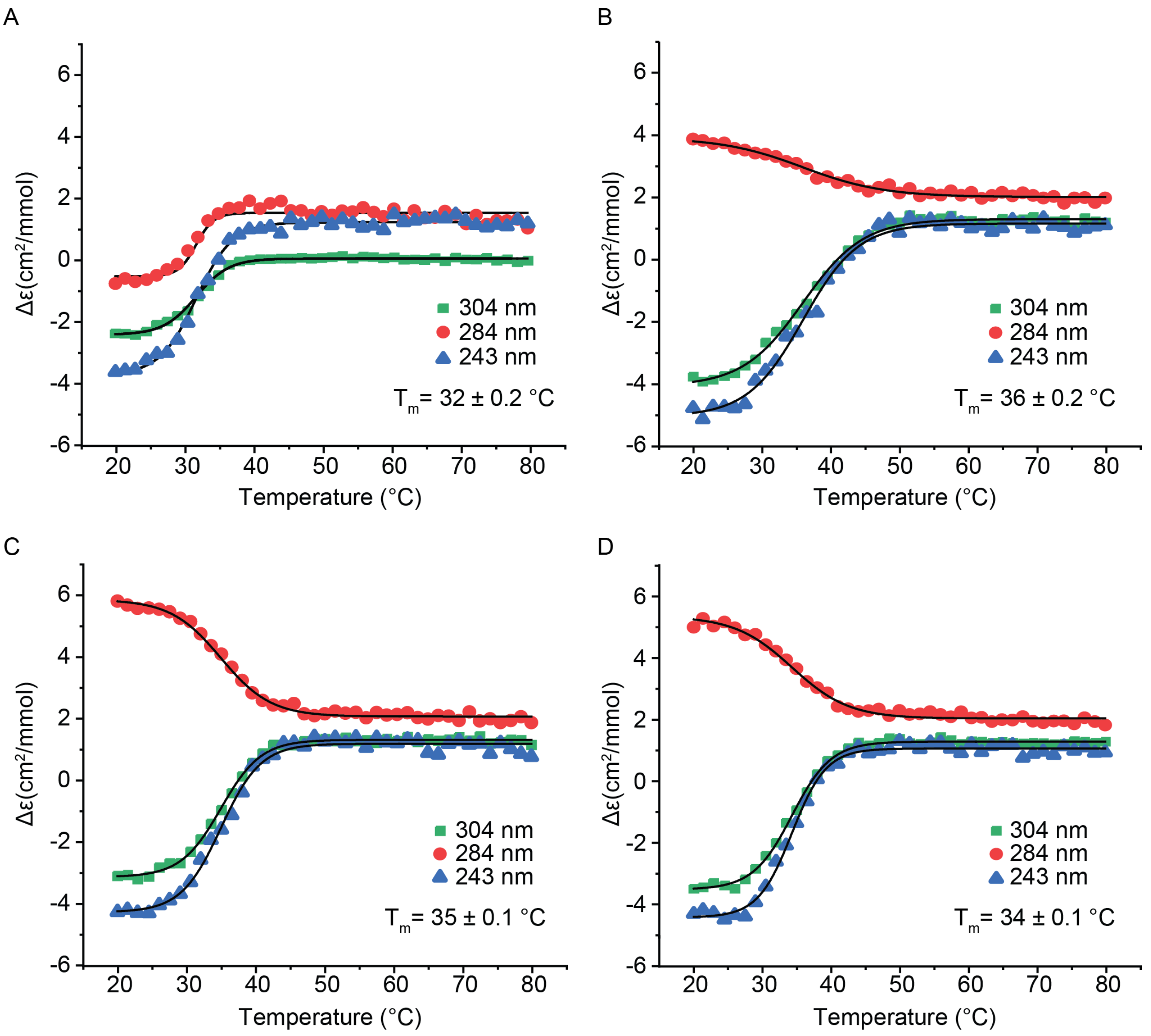

Supplemental Figure 2. CD monitored thermal denaturation of (A) (GA)12, (B) U2A, U8A, U14A, U20A, (C) U4A, U10A, U16A, U22A and (D) U6A, U12A, U18A and U24A. All data were fit to the Boltzmann equation to determine the T_m.

Supplemental Figure 3

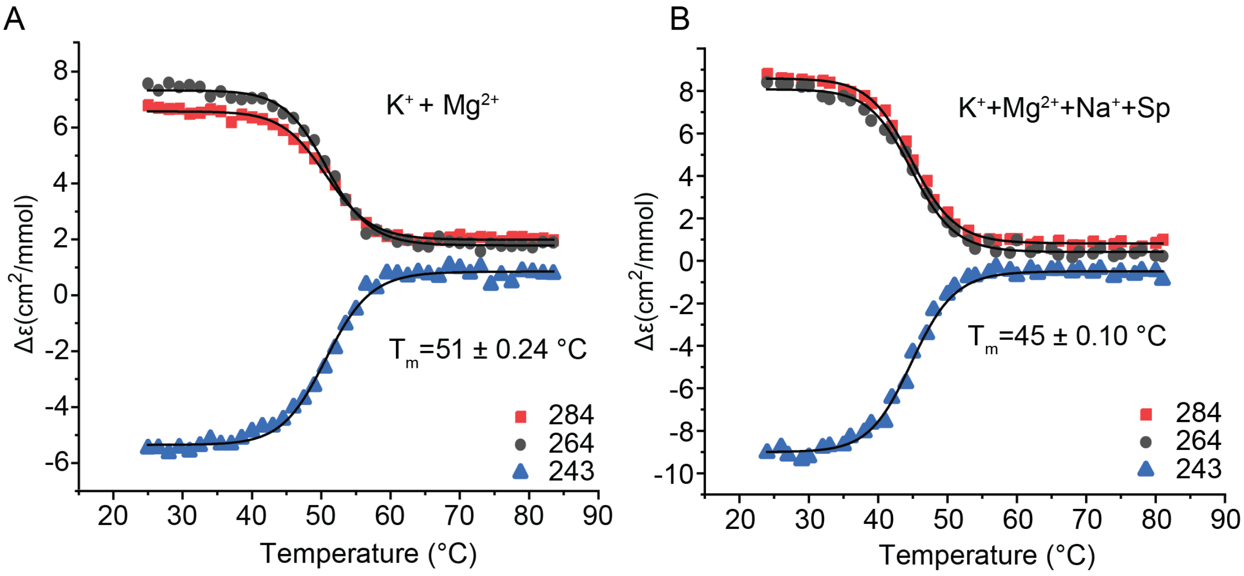

Supplemental Figure 3. (B) Thermal melt of $(GU)_{12}$ in 150 mM K^+ and 2 mM Mg^{2+}. (C) Thermal melt of $(GU)_{12}$ in K^+, Na^+, Mg^{2+}, Sp buffer (140 mM KCl, 10 mM NaCl, 2 mM $MgCl_2$, 0.3 mM spermine and 0.4 mM spermidine).

Supplemental Figure 4

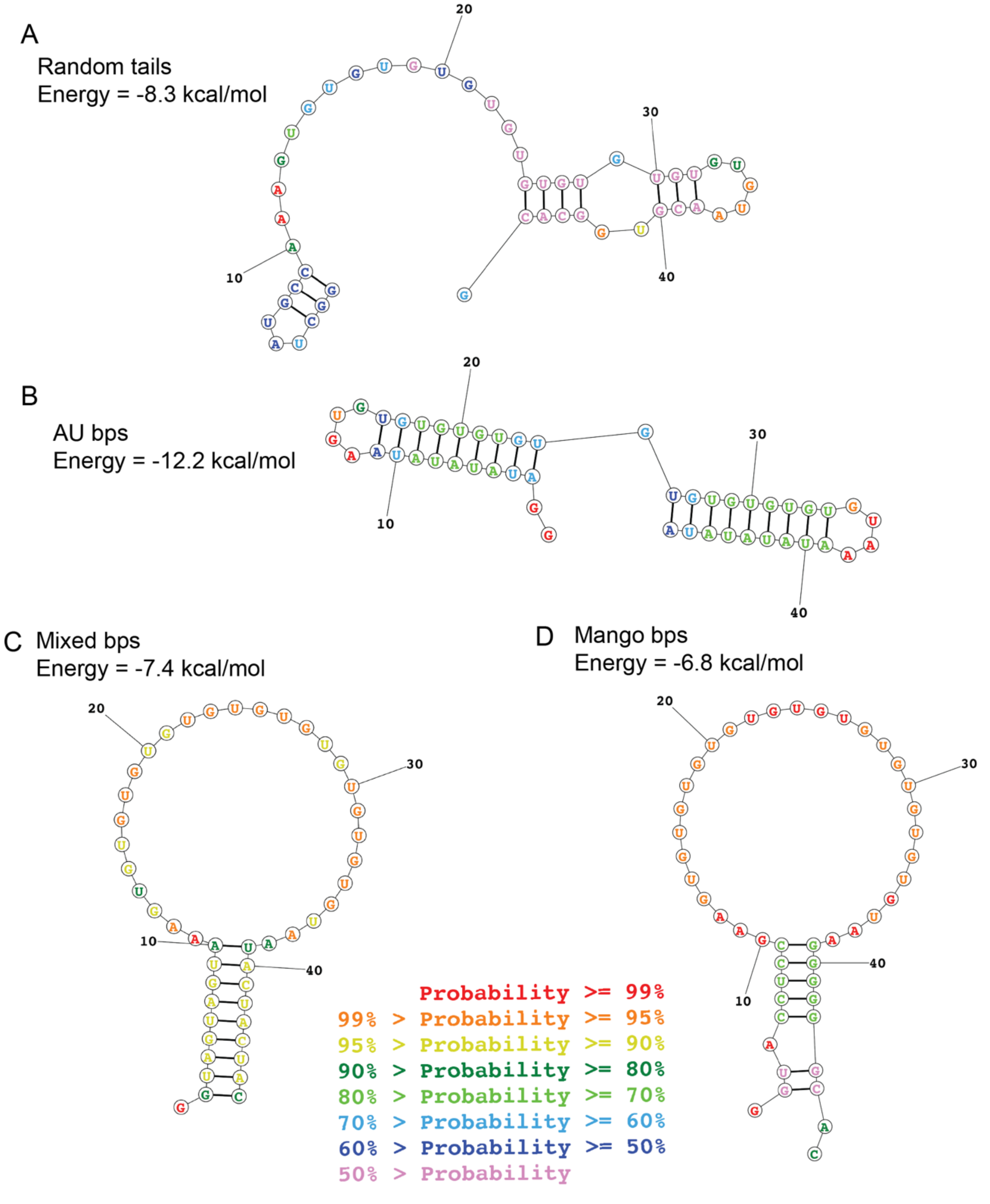

Supplemental Figure 4. Predicted secondary structure free energies and base pairing probabilities for 47 nucleotide RNAs with the indicated 5′ and 3′sequences flanking $(GU)_{12}$.

Supplemental Figure 5

A U to N single nucleotide substitutions

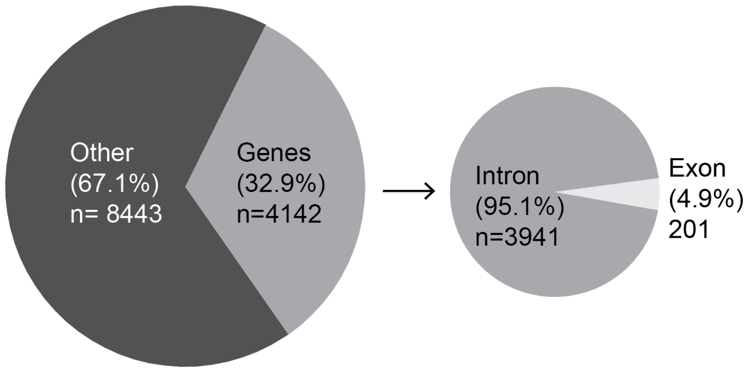

B 2-4 U to A substitutions

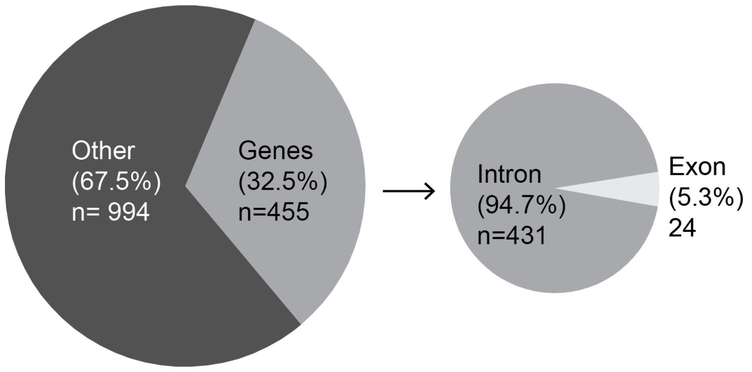

C $(GA)_R$, R ≥ 11.5

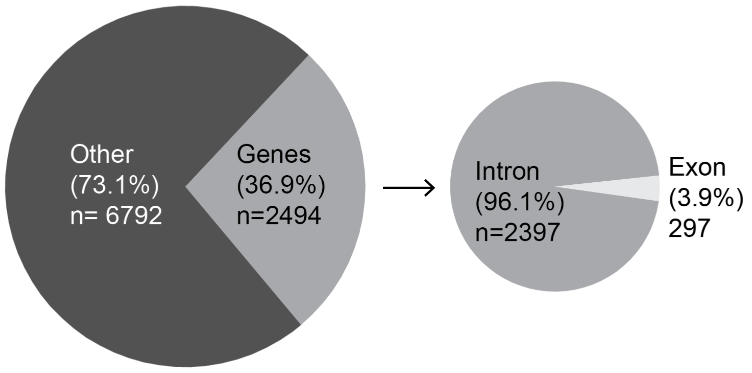

Supplemental Figure 5. Number and distribution of potential pUG fold sequences containing (A) single nucleotide U to N (where N is any nucleotide) substitutions, (B) 2-4 U to N substitutions, and (C) 11.5 or more GA repeats in the human genome.
